# Multiple gastric adenocarcinoma of the fundic gland type with various endoscopic features in autoimmune gastritis: a case report and literature review

**DOI:** 10.1093/gastro/goaf066

**Published:** 2025-09-15

**Authors:** Xianhong Zhao, Zhifa Zhang, Zhihua Du, Xiaohua Du, Wenbin Wu, Haiyan Zhang

**Affiliations:** Department of Gastroenterology, The Second Affiliated Hospital of Guangzhou University of Chinese Medicine, Guangzhou, Guangdong, P. R. China; Department of Pathology, The Second Affiliated Hospital of Guangzhou University of Chinese Medicine, Guangzhou, Guangdong, P. R. China; Department of Pathology, The Second Affiliated Hospital of Guangzhou University of Chinese Medicine, Guangzhou, Guangdong, P. R. China; Department of Pathology, The Second Affiliated Hospital of Guangzhou University of Chinese Medicine, Guangzhou, Guangdong, P. R. China; Department of Gastroenterology, The Second Affiliated Hospital of Guangzhou University of Chinese Medicine, Guangzhou, Guangdong, P. R. China; Department of Gastroenterology, The Second Affiliated Hospital of Guangzhou University of Chinese Medicine, Guangzhou, Guangdong, P. R. China

## Introduction

Gastric adenocarcinoma of the fundic gland type (GA-FG) is an epithelial tumor characterized by low-grade atypia and differentiation toward fundic glands. GA-FG lesions have been reported to be covered by normal foveolar epithelium and arise from the non-atrophic mucosa of the upper or middle stomach [1]. At the same time, these lesions originate from the deep layers of the gastric mucosa and can be macroscopically observed with diverse various endoscopic features, such as a submucosal tumor (SMT)-like morphology. Here, we present a rare case involving nine GA-FG lesions with varying endoscopic features in autoimmune gastritis (AIG).

## Case report

A 50-year-old man underwent gastroscopy at another hospital as part of a health checkup. Pathological analysis of one biopsy revealed oxyntic gland adenoma. For further evaluation and treatment, he presented to our center. A carbon-13 urea breath test was negative. The patient had no history of long-term proton pump inhibitor use. He tested positive for anti-parietal cell antibodies, while all other blood test results were within normal limits. Additionally, an abdominal computed tomography scan showed no abnormalities.

**Figure 1.**
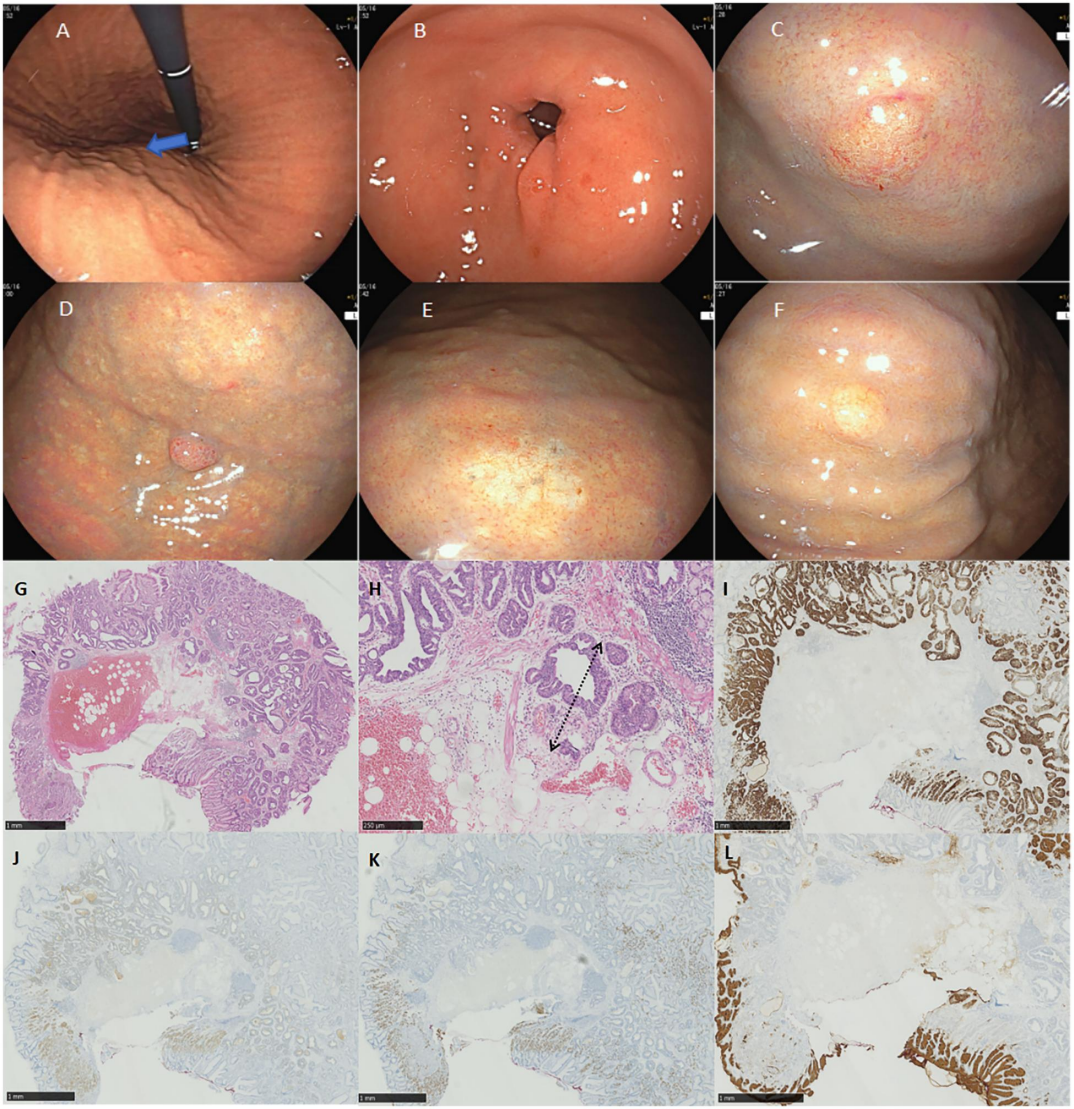
Characteristics of endoscopy and histopathological evaluation of lesion type two. (**A**) bamboo joint-like appearance aligns in rows along the long axis of the gastric corpus folds (bold arrow). (**B**) The antrum does not show the typical image of atrophy. (**C**) Lesion type one presents as a slightly reddened surface with Paris Classification of type 0–IIa, measuring 8 mm in diameter. (**D**) Lesion type two presents as a reddened surface with Paris Classification of type 0–Is, measuring 8 mm in diameter. (**E**) Lesion type three presents as a whitish surface with Paris Classification of type 0–IIb, measuring 10 mm in diameter. (**F**) Lesions type four are identified as SMT-like shape with tree-like appearance vessels on the surface, measuring approximately 5–8 mm in diameter. (**G**) Histopathological appearance of the main lesion. (**H**) Vertical depth of invasion of the tumor (the submucosa) is 488 μm. The black arrow indicates the distance from the muscularis mucosae to the submucosal invasion. (**I**, **J**) MUC6 (I) and Pepsinogen I (J) are diffusely positive in the tumor glands. (**K**) H^+^/K^+^-ATPase is distributed in the vicinity of the glandular neck. (**L**) MUC5AC is strongly expressed in the foveolar epithelium-like cells in the surface layer.

Gastroscopy revealed shallow folds extending across the entire area of the greater curvature of the gastric corpus. Nodular manifestations aligned in rows along the long axis of the gastric corpus folds showed a bamboo joint-like appearance (Figure 1A) [2]. The antrum did not display the typical appearance of atrophy (Figure 1B). Nine lesions with diverse endoscopic characteristics were identified. Based on macroscopic morphology, these lesions were classified into four types. Type 1: located in the gastric fundus, this lesion presented as a slightly reddened surface and was categorized as Paris Classification type 0–IIa, with a diameter of 8 mm (Figure 1C). Type 2: this lesion was located in the anterior wall of the upper third of the gastric corpus, presenting as a reddened surface with Paris Classification type 0–Is and measuring 8 mm in diameter (Figure 1D). Type 3: found on the greater curvature of the upper third of the gastric corpus, this lesion displayed a whitish surface and was categorized as Paris Classification type 0–IIb, with a diameter of 10 mm (Figure 1E). Type 4: comprising six additional lesions, this group was scattered throughout the middle and upper third of the gastric corpus, measuring 5–8 mm in diameter. These lesions exhibited an SMT-like morphology with tree-like vessels visible on their surfaces (Figure 1F). Based on the endoscopic characteristics and previous biopsy results, these lesions were tentatively diagnosed as GA-FG, necessitating differentiation from neuroendocrine tumors (NETs). An en-bloc resection of all lesions was performed via endoscopic treatment.

Histopathological examination revealed mild-to-moderate lymphocytic cell infiltration between the gastric oxyntic glands. Tumor glands, replacing the fundic glands and covered by a normal foveolar epithelium, showed marked atypia in the deeper layers. These tumor glands had deeply invaded the muscularis mucosae, reaching the submucosal layer (Figures 2G, H). The abnormal tumor glands predominantly displayed an “endless glands” pattern. Prominent atypia and mitotic figures were not observed in the tumor cells. Immunohistochemically, the tumor glands were positive for pepsinogen-I and MUC6, but negative for H^+^/K^+^-ATPase and MUC5AC (Figures 2I–L). Ultimately, the nine lesions were diagnosed as the chief cell predominant type of GA-FG.

The patient has undergone two follow-up visits, including gastroscopy. The wound had already formed scars, with no local recurrence. Additionally, the computed tomography scan showed no positive findings.

## Discussion

Our case demonstrated nine GA-FG lesions with diverse endoscopic characteristics in patients with AIG. Several distinctive features were observed. First, inducing GA-FG from the residual fundic glands in AIG appeared to be challenging. Second, nine tumor lesions with diverse endoscopic characteristics posed diagnostic and therapeutic challenges for endoscopic physicians.

Common lesions in patients with AIG include type 1 NETs, residual oxyntic mucosa pseudopolyps, hyperplastic polyps, and pyloric gland adenomas [3]. However, due to the progressive loss of gastric fundic glands, cases of GA-FG in patients with AIG are few. Wang *et al.* [4] reported a case of GA-FG mixed with a well-differentiated NET in a patient with AIG. The prominent lesion was located in the upper third of the gastric corpus and exhibited branching dilated blood vessels alongside superficial ulceration on its surface. Similarly, Sumida *et al.* [5] described a case of GA-FG in a patient with AIG. The flat, elevated lesion was located in the middle third of the gastric corpus and was treated via endoscopic submucosal dissection. Although previous research has shown that an independent histological assessment detects no background atrophy in nearly all of 340 GA-FG lesions [6], it is plausible that the reduced number of fundic glands in AIG may also create conditions conducive to the development of GA-FG. AIG is categorized into three stages—early stage, florid stage, and end stage—based on histological features [7]. In the early stage, despite the obscured two-layered structure of the parietal and mucous neck cell layers and the chief cell layer, the ratio of the length of the gastric pit to that of the gastric gland in the fundic mucosa remains normal. This may serve as a prerequisite for the development of GA-FG.

The endoscopic characteristics of GA-FG are primarily described as SMT-like, with a tree-like vessel appearance on the surface. Zhai *et al.* [6] analyzed the endoscopic features of 328 patients with 340 GA-FG lesions, reporting that most cases involved a single lesion, while several cases showed multiple lesions. The guanine nucleotide-binding protein alpha-stimulating complex locus mutation has been previously identified as a characteristic genetic feature of GA-FG [8]. However, no studies to date have investigated whether multiple lesions are associated with genetic abnormalities, highlighting the need for further genetic analysis in future research. Regarding treatment, although small GA-FG lesions can present submucosal infiltration, they generally lack tendencies for lymphovascular invasion or distant metastasis [9]. Imamura *et al.* [10] reported a rare case of a patient with GA-FG lesions. The largest lesion was treated with endoscopic submucosal dissection and the patient was followed up with biannual endoscopy and computed tomography because he refused further surgical interventions. Over a 5-year follow-up period, no tumor growth or metastasis was observed [10]. To optimize the postoperative quality of life, en-bloc resection of all lesions was performed by using endoscopic treatment. One year post-operation, no tumor recurrence or metastasis was detected.

In conclusion, we presented a case of nine GA-FG lesions exhibiting various endoscopic features in AIG. We hope that our case contributes to a deeper understanding and broader insights into these conditions.

## Authors’ contributions

X.H.Z. and H.Y.Z. conceived and designed the report. Z.F.Z. and Z.H.D. collected the data. X.H.Z. reviewed the literature and wrote the manuscript. X.H.D. and W.B.W. revised and edited the manuscript. All authors read and approved the final version for submission.
